# Administrative data analysis of student attrition in hungarian medical training

**DOI:** 10.1186/s12909-022-03276-z

**Published:** 2022-04-26

**Authors:** Gabriella Pusztai, Zsuzsanna Demeter-Karászi, Emese Alter, Rita Marincsák, Ilona Dóra Dabney-Fekete

**Affiliations:** 1 Institute of Educational Studies and Cultural Management, University of Debrecen, Debrecen, Hungary; 2 Faculty of Dentistry, University of Debrecen, Debrecen, Hungary

**Keywords:** Medical training, Dropout, Delayed graduation, Form of finance, International students

## Abstract

**Background:**

There is numerous empirical evidence supporting that college students studying in fields with rigorous curriculum and high requirements, such as medical training, are characterized by a higher risk of attrition than their peers. Since Hungarian medical training attracts more and more international students every year, the issue of dropout can have a global impact. Our study aimed to examine attrition risks of local and international students in Hungarian medical training.

**Methods:**

In our study, we examined the dropout behaviour of all medical students who started their studies in 2010 in Hungary (*N* = 2391) by analysing longitudinal administrative data of those who studied between 2010 and 2017. Doing this, we conducted descriptive statistics and uncovered the risks of dropout using binary logistic regression.

**Results:**

Our results indicate that the danger is primarily increased by factors directly linked to or indicating poor academic performance (slow pace of credit accumulation, tuition-based forms of finance). Individual characteristics, namely gender, and citizenship, also have a moderate but significant effect on the latter.

**Conclusions:**

Thus a policy proposal can be formulated consisting of making the training network less rigid, devoting more educational attention to and providing targeted mentoring for students with learning difficulties and academic hardships. Foreign medical students studying in Hungary comprise a large group that has a high attrition rate, making it a prime target for dropout-reducing programs.

## Background

### The main theories of dropout

College dropout is one of the most researched topics in the field of education. The reason behind the scientific interest in this topic is due to the national and individual significance of attrition. While the expansion made higher education accessible to a wide range of students, it also led to several new challenges that universities had to face. For national governments and policymakers, it became increasingly important to have proof that investing in tertiary education is profitable for the state. According to the human capital theory, funding higher education is a profitable state investment, not only because those with a college degree tend to be more productive in the labour market, but also due to its positive externalities, for example higher employment rates, a more adaptable workforce, public spending cuts, low crime rates, etc. [1–3]. It is also clear that investing in tertiary education is only profitable when those in the system leave college with a degree. Research of the sheepskin effect, which represents the added value of getting a higher education degree [4], has shown that graduates of all types and fields of higher education have higher wages than those with a secondary level school-leaving certificate [5]. This means that college attrition is a waste of the time and money that the students and their families invested in their education from an individual perspective and also as an unprofitable investment from the government’s perspective.

The first complex theories of attrition were born in the 1970s and relied on Durkheim’s theory of suicide [6]. Spady [7, 8] theorized that poor performance and inconsistent relational support led to attrition, while the decision-making process behind it is influenced by the interactions between the students’ dispositions, interests, attitudes, skills, expectations, and the institution’s courses, faculty members, administrators, and peers. Grades, intellectual development within the academic system, support from friends and peers, and normative congruence within the social system are the main factors of dropout, meaning that academic and social factors both have a significant effect.

Tinto’s theory of attrition [9, 10] is one of the most influential concepts on dropout and higher education research. Tinto’s work emphasized persistence and its role in attrition. According to him, persistence can be understood as an outcome of personal characteristics and a dual commitment to the institution and to college completion. This is heavily dependent on how integrated a student is into the institution’s academic and social systems. Therefore, based on Tinto we can argue that the prime predictor of academic outcome is the completion of college courses and the commitment to obtain a degree.

Conversely, Bean [11, 12] built his theory on labour turnover, understanding college attrition to be a phenomenon similar to leaving a workplace. In Bean’s theory dissatisfaction is an important factor in both cases, and Grade Point Average (GPA), development, institutional quality, and practical values play a role in college attrition that is parallel to the impact of salaries in employee turnover [13].

Even though the above-presented theories of attrition were efficient in predicting traditional student dropout, the factors influencing this behaviour in non-traditional students only came to the forefront of research in the 1980s. According to Bean and Metzner [14], non-traditional students (usually coming from disadvantaged backgrounds) are more affected by external factors such as responsibilities, environment, and finances, than their peers with a more beneficial background. Cabrera, Nora, and Castaneda [15] have also concluded that environmental factors might have a stronger impact on dropout than Tinto’s model implied [13].

### Dropout among medical students

Even though medical students’ attrition is primarily based on the factors described above, it may be that medical students are more heavily affected by monetary and academic factors specific to that field than students of other fields. These attributes contributing to dropout in medicine are high academic requirements (both upon entry and in order to graduate), the long training duration, and the infrastructural demand of training professionals. Based on the literature, the most important factors of attrition in medical training are: academic preparedness, professional socialization, curriculum and credit accumulation, passive semesters, and form of finance.

### Academic preparedness

Since medical training sets high academic demands for students, academic preparedness has a major influence on dropout in this field. High school results, and the year of admission are proved to be good predictors of students’ success in obtaining a degree, while entrance exam scores indicated whether an individual would complete their studies without exceeding the period specified in the curriculum [16].

### Professional socialization

According to previous findings [17, 18], insufficient professional socialisation can result in dropout from medical courses and medical professionals quitting the field, causing serious labour shortages in the healthcare system. The early formation of professional identity, which can limit the rate of student dropout, is strengthened by attachments to disciplinary values and relationship networks, by high admission requirements and favourable career possibilities [19, 20].

### Curriculum and credit accumulation

Medical training is very prestigious worldwide, thus it has rigid selection processes. Selecting students with high persistence and performance can be done by setting high requirements during the admission process, but it is also possible to further select students with the best performance by setting high output standards later. This high bar can be set through training courses comprised of several prerequisites. Some of these prerequisites are called, “bottleneck courses” due to their high failure rates. These rates in turn make these courses heavily influence on-time graduation and attrition [21]. Therefore the pace of credit accumulation can be an indicator of the students’ ability to pass bottleneck courses and successfully obtain a degree.

### Passive semesters

The pace of credit accumulation is not the only factor of academic progression connected to the risk of attrition. The plausibility of dropout is also increased by the number of passive semesters (when the student does not formally participate in higher education) [22]. In light of Tinto [9, 10], passive semesters can weaken the process of social and academic integration into the institution, and also can indicate a decrease in the students’ commitment to obtain a degree.

### Form of finance

The cost of higher education and threat of potential student debt can also grow the risk of dropout, especially in fields with high tuition fees. According to Doll et al. [23], institutional factors of dropout usually lead to the student being pushed out of education. In this section, we are focusing on tuition fees and indebtedness. First of all, there are three main categories of tuition fees: 1. tuition fees for all (upfront/deferred), 2. dual-track tuition fees, and 3. no tuition fees [24]. The Hungarian tuition system is closest to the, “dual-track” system, meaning that the top performers can be state-funded, while others have to pay their way either out of pocket or by government-subsidized loans.

In the West, there is significant indebtedness among novice doctors deriving from tuition fees. Even though indebtedness is a challenge in the higher education system as a whole, it is even more troubling in the field of medicine. This is due to higher tuition fees compared to other fields and because earning a professional income here requires more time spent training [25]. This excessive amount of study time can also harm students’ mental health. All in all, dropout is a severe burden on public resources and a great detriment to individual resources.

### Hungarian tertiary education and medical training

#### The structure of medical training

Unlike other areas at the turn of the millennium, following the Bologna procedures, no multi-cycle courses were introduced in Hungarian medical training. So, for example, while 3 + 2 year BA/BSc^1^ and MA/MSc^2^ courses were introduced in the field of economics, the one-cycle educational structure was kept in the medical, legal, pedagogical, and theological fields. This means that future doctors have to study for 6 years to obtain a degree.

Regarding the admission process and the requirements for the completion of courses, medicine is highly selective in comparison with other fields in the Hungarian higher education system.^3^ Medical students start their internships and residency years after obtaining their degree so that they can take their medical specialist exam at the end of their 3–5 year long courses. This extremely long period of study (6 years in college + 3–5 years as residents) keeps medical students from becoming self-sufficient and starting their own family. In comparison, obtaining a degree in STEM fields, economics, social sciences, and teacher training makes it possible to reach a favourable position in the labour market without further education. Compared to those living and working in Western countries, Hungarian medical professionals are also underpaid. To be able to make a comfortable living, Hungarian doctors usually need to take second jobs, which can cause burnout. The slow pace of obtaining a degree causes labour shortages, which are further exacerbated by students quitting, moving abroad, and by the aging of the medical society left behind [17, 26].

#### Different forms of funding

In Hungarian medical training, based on funding, there are 3 groups of students. 1: Students with Hungarian citizenship are financed by the state if they have reached the required scores at admittance and if the period of their studies has not exceeded 7 years. Only some Hungarian students who started their second degrees or who are not state-funded complete their studies in self-financed courses.^4^ 2: Most foreign students from European and Asian countries study in self-financed courses in English or German.^5^ 3: The last group includes foreign students from developing countries whose studies are financed by the Hungarian state through the Stipendium Hungaricum Scholarship.

The contemporary relevance of our research is supported by the fact that in 2015, a new system of financing was introduced for Hungarian students who had been studying free of charge up to that point. This new system monitors the study performance and grades of students annually, and those whose grade-point averages do not reach 3.00 (rated on a 5-point scale), and who do not get at least 36 credits yearly, lose their state-funded status and have to then pay tuition fees. Considering that the average household net adjusted disposable income per capita in Hungary is 18430 USD and the OECD average is 33604 USD [27], the yearly expense of 8000 USD can lead to significant growth in dropout.

In 2012, public funding for the first degree was also debated. Due to increasing international mobility, the lack of doctors in Western Europe, and the desire to gain experience and have better earnings, many young doctors in Hungary moved abroad right after graduating. As this can also be interpreted as a situation in which the return of the Hungarian state’s investment in human capital was received by other states, the Hungarian government decided that investing in every applicant’s studies without any kind of selection was not profitable. In 2012, student contracts were introduced, which stated that after obtaining state-financed degrees, students would have to remain in the country and work for a certain period. Then, in the middle of the decade, the ministry for higher education decided that to reduce the burden on the state budget, students who cannot meet certain requirements should cover their study expenses on their own. To make covering tuition easier, it was made possible to pay fees on credit. Medical training, consequently, was placed on the market for certain groups. Though this well-meaning move appears nothing short of benign and helpful, it can lead to the accumulation of huge debts. Considering this, students who would get better grades in the next semester may choose to drop out, so that they can avoid becoming indebted.

#### The internationalization of Hungarian medical training

In Hungary, approximately 30% of students are from foreign countries [28], most of whom are studying medicine [29]. To understand why these foreign students decide to study in Hungary, we need to distinguish between the two main groups of foreign students in Hungarian medical training. The first group contains mostly students coming from Western Europe and Scandinavia (Germany, Norway, Sweden), while participants of the second large group are coming from non-European, oftentimes developing countries (Iran, Israel, Turkey, Vietnam, Nigeria, South Korea, Japan) [29]. For students from Western Europe and Scandinavia, relatively low tuition fees, low costs of living [30, 31], and in some cases (e.g. Germany, where admission requirements are very high) lower admission requirements might play the biggest roles in their decision to choose a Hungarian higher education institution. In contrast, students coming from non-European countries might consider that the costs of studying in Hungary are a lot lower for them than for others, thanks to scholarships that are only available for students from outside Europe (e.g. the Stipendium Hungaricum Scholarship). These students also often perceive obtaining a degree in a European country as a starting point for their global mobility [32].

#### The status and difficulties of foreign students in Hungarian higher education

According to the international literature, students studying in foreign countries usually face more difficulties than their local peers. These hardships can come from multiple sources, primarily cultural barriers, lack of social support, homesickness, discrimination, and prejudice [33]. On the one hand, previous research dealing with international student satisfaction with their studies in Hungary indicates that these students are generally content with the study environment, the services offered to them, and the wide range of intercultural activities [31]. On the other hand, because the foreign language proficiency of local students is poor and they generally do not want to make international friends, foreign students find it hard to get to know their Hungarian peers. Thus, in an effort to curb dropout among international students, social integration and support are made available to all foreign students.

### Hypotheses

Based on the literature, in our hypotheses, we focused on the role that the form of funding, passive semesters, the pattern of credit accumulation, and being an international student potentially play in attrition.

- H1. The form of finance plays a significant role in student dropout: dropout rates are lower among those who study in state-financed form compared to their peers who carry out their studies in self-financed form.
- H2. The patterns of credit accumulation can be significant predictors of dropout: dropout rates are higher among those students who collect credits at a slower pace than in the case of those that collect the expected number of credits.
- H3. The number of passive semesters significantly increases the probability of attrition: those students who have more passive semesters are characterized by a higher risk of dropout than those who do not pause their studies.
- H4: We hypothesize that foreign students drop out more frequently than Hungarian students.

## Methods

### Database

Our research aimed to examine the dropout behaviour of Hungarian and foreign medical students. In this study we analysed data from an anonymized individual higher education database provided by the Hungarian Educational Authority, accumulated and stored by the Hungarian Higher Education Information System. In this analysis, we focused on the progress of students from four Hungarian universities who started their studies in 2010. The database included longitudinal administrative data between 2010 and 2017, which provided us with valid information on the progress of these students.

### Variables used in the analysis

During the analysis we worked with the following variables: gender, number of passive semesters, financing, pace of credit accumulation and a binary nominal variable based on the first citizenship of the individual (individuals with Hungarian citizenship are referred to as local students, while those with any other first citizenship are in the group of international students).

### Statistical analysis

During our research, we used descriptive analysis, in which we examined and compared dropout rates in certain groups. To explore the reason(s) behind dropout, we conducted binary logistic regressive analysis and we applied the Forward Wald method. We regarded *p* < 0.05 as a significant result. Analyses were done with the IBM SPSS Statistics 25.0 program package (SPSS, Chicago, IL).

### Sample

Our sample consisted of 2391 participants, 40% of whom were Hungarian citizens (Table 1.). The largest proportions of foreign medical students were from Germany (22.3%), Israel (5.1%), Norway (4.7%), Spain (3.8%), Nigeria (3%), South Korea (2.8%), and Sweden (2.5%). As Table 1 shows, most students belonged to the self-financed category throughout their studies. It is important to note that the proportion of international students starting their studies state-funded was 1.6%, while the local subgroup boasted 92.2%.

**Table 1:** Participants distribution by nationality, and form of finance

		State-financed	State-financed then shifted to self-financed	Self-financed then shifted to state-financed	Self-financed
Form of finance	N	820	86	33	1452
	%	34.3%	3.6%	1.4%	60.7%
		Hungarian		Foreign	
Nationality	N	957		1434	

Source: Higher Education Information System Hungary, Hungarian Educational Authority, own edition

## Results

**Fig. 1:**
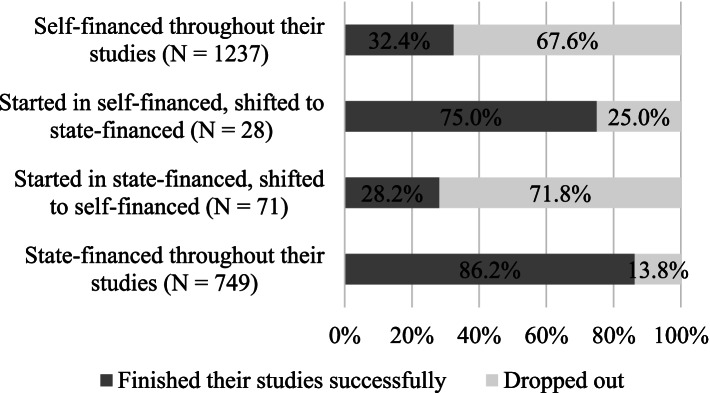
Attrition rates and different forms of finance of the whole sample (*N* = 2085)

To test our first hypothesis, we analysed the dropout rates among groups with different forms of finance. Accordingly, we conducted the analysis of the Hungarian subgroup separate from the foreign subgroups. As you can see in Fig. 1, dropout rates are the highest among those, who shifted from a state-funded to a self-financed status (71.8%) and among those who were paid out of pocket throughout the duration of their studies (67.6%). On the contrary, only 25% of self-financed-to-state-funded students dropped out, and 13.8% quit school who were state-funded throughout their studies.

**Fig. 2:**
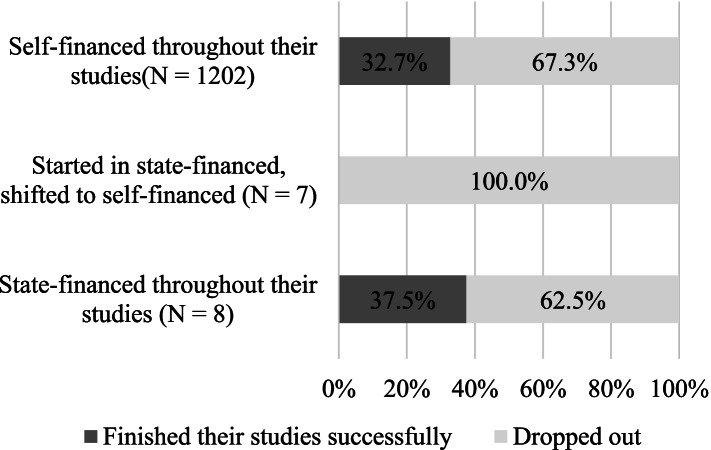
Attrition rates and different forms of finance among international students (*N* = 1217)

**Fig. 3:**
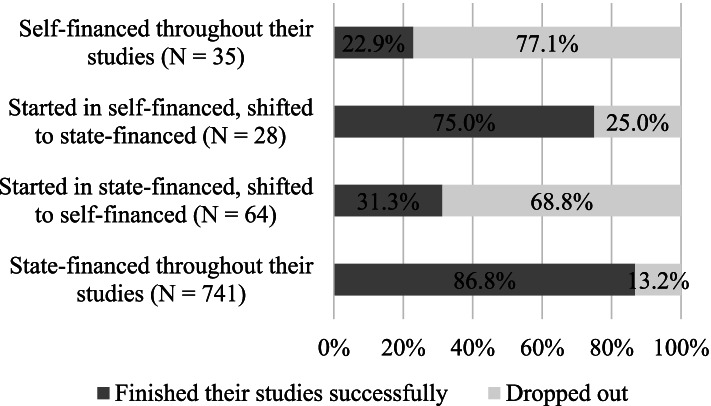
Attrition rates and different forms of finance among local students (*N* = 861)

When we compare the data of Hungarian and foreign students (Fig. 2 and Fig. 3), we can see that most international students were in the self-financed group throughout their studies, and their dropout rates are correspondent to this: 100% of those, who were shifted to self-financed status from the state-financed one dropped out. The attrition rates among state-financed students were similar to the self-financed. In the Hungarian subgroup however, being shifted from state-financed to self-financed appears to increase the chance of dropout significantly. While 13.2% of those who were state-financed throughout their studies dropped out, dropout rates were 68.8% in the case of those who started their studies in state-financed form and were later shifted to self-finances. Dropout rates were highest among the self-financed (77.1%), a stark contrast to the lower number of those who became state-funded later (25%).

To test our hypothesis concerning the role of passive semesters, credit accumulation, form of finance, and being an international student in attrition, we used a binary logistic regression model. The dependent variable was dropout (dropped out or not), while as background variables we considered the following: gender, age, number of passive semesters (less than 2/2 or more), form of finance at the beginning of studies (self-financed/state-financed), and citizenship (Hungarian/foreign). By applying the Forward Wald method, we obtained a model in which gender (male) and citizenship (foreign) slightly increased the chance of attrition. Having passive semesters affected attrition very little, while self-financing and slow credit accumulation drastically increased the risk of dropout (Table 2).

**Table 2:** Model 1. Results of the binary logistic regression

Variables	B	S.E	Wald	df	Sig	Exp(B)
Gender^a^	.244	.118	4.3	1	.038	1.277
Citizenship^b^	.363	.32	1.285	1	.257	1.438
Passive semesters^c^	-2.245	.69	10.59	1	.001	.106
The pace of credit accumulation^d^	3.786	.231	268.054	1	< 0.001	44.099
Form of finance^e^	1.771	.319	30.862	1	< 0.001	5.879
Constant	-2.205	.125	312.039	1	< 0.001	.110

Source: Higher Education Information System Hungary, Hungarian Educational Authority, own edition

^a^0 – Female, 1 – male.

^b^0-Hungarian, 1- Foreign

^c^0-Less than 2,1-2 or more

^d^We coded the pace of credit accumulation by the number of credits gained by the student at the end of the fourth semester: 0 – The student gained at least 60% of the suggested number of credits specified by the institution (120 credits), 1 – The student gained less than 60% of the suggested number of credits.

^e^0- State-financed,1 -Self-financed

We also compared those who completed their studies successfully with the dropouts from the Hungarian and international subsamples. As you can see in Table 3, almost half of those who started their studies in 2010 did not obtain a degree (47.8%). The dropout rate between international students and local ones is staggering—with 67.5% being international and 20.3% being local. Most foreigners were self-financed, so it is possible that beyond cultural differences, financial difficulties could explain this difference.

**Table 3:** Dropout rates among local and international students

	**Local**	**Foreigner**	**Total**
Completed their studies successfully	79.7%	32.5%	52.2%
Dropped-out	20.3%	67.5%	47.8%

Source: Higher Education Information System Hungary, Hungarian Educational Authority, own edition

## Discussion

Even though the internationalization of medical training is not a new and unexpected phenomenon, the Hungarian research literature barely deals with the topic of attrition among international students. It would be worthwhile however, since 60% of medical students in Hungary are foreign students. Stemming the tide of dropout and successfully integrating these students would leave an international footprint. Unfortunately, in accord with most attrition theories and previous research, international students cannot integrate as easily as their peers, and this obstacle makes them more likely to drop out. It is a multi-faceted issue. These students live far from their parents, friends, and significant others, they have no social support to help cope [34] with stressful life situations, making friends is hindered by cultural and linguistic barriers. Added up, all this makes students feel even more isolated [31].

Our hypotheses are expectations about empirical associations without causal claims, since we have examined neither the structure of the causal chain, nor the fruition of the causal criteria. In the first three hypotheses of our study, we focused on the academic factors of dropout. We assumed that academic performance, self-financed tuition, more passive semesters, and slow credit accumulation can significantly predict the risk of attrition. According to our results, the pace of credit accumulation proved to have the strongest association with attrition. This finding is in line with previous literature. A selective institutional approach is a factor of dropout specific to medical training that can lead to pushing students out of the program [23, 35]. Students can easily be discouraged from continuing their studies when they are confronted by high academic requirements, numerous prerequisite courses, and repeated failures [35].

Our results, which correspond with previous findings, have also supported our hypotheses regarding self-financed status. Special literature highlighted that high tuition fees can be negative institutional factors that force students out of higher education [23, 35]. Tuition fees in Hungarian medical training are relatively high. Therefore, students with academic difficulties and those who fail key “bottleneck courses” think twice about drawing out their studies. In the case of Hungarian students, starting out self-financed or getting shifted from state-financing correlates with lower academic performance, directly the funding to dropout. In contrast, most international students started their studies with self-financed status regardless of their entrance exam scores. Thus, for them high tuition fees might be that dropout catalyst.

With other variables included in our logistic regression model, the role of passive semesters did not prove to be as important as shown in previous research [35, 36]. A possible explanation of this finding is that the student deciding to not participate in higher education is more an indicator of poor integration, dissatisfaction with training, or low persistence than a factor that itself leads to attrition.

During our research, we put great emphasis on examining the position of international students in Hungarian medical training. Our analysis showed that attrition rates are significantly higher in the subsample of international students than in locals. Since most international students were self-financed, we also examined finance as a dropout factor in the Hungarian and international subsamples. High tuition rates were also scrutinized, appearing as a factor among international students. If these students were state-financed, as most Hungarian students are, they probably would dropout less frequently. The results of logistic regression have also shown that being an international student significantly increases the risk of attrition, though its effect is relatively weaker in comparison to other examined factors.

## Conclusions

Overall, studying the phenomenon of dropout is an important issue for the higher education system as a whole. Providing state-financed higher education is costly for the state but if students drop out, it can be extremely wasteful, on both the societal and individual levels. The chances of dropping out are increased by study-related (slow pace of credit accumulation, passive semesters), finance-related factors, and by a person’s nationality. Medical students usually enter higher education with high performance and a dedicated attitude, but despite this, only half of the students who had started their studies in 2010 were able to graduate by 2017.

It is important to note, our study was limited. The database we used provided information regarding the progression of only one sample, and we only had access to specific academic and administrative data. We could have made further important connections by examining academic grades, but based on the database we can only draw conclusions on unsuccessful course performance. However, if we interpret our results in light of Tinto’s theory of attrition [9, 10], we can conclude that lack of integration can be identified based on administrative data only. Since institutions can see the data we have collected, it can aid the early recognition of students with a high risk of attrition, allowing institutions to give them targeted support.

Previous research focusing on international students studying in Hungary has already put great emphasis on exploring foreign student satisfaction with their studies. While these results have significantly contributed to our knowledge about the position of international students and their specific difficulties, their academic progress is an important research topic that has not yet come to the forefront of educational research in this region.

Beyond taking a step forward to understand the phenomenon of attrition, the results of our study have implications for the internationalization of medical training as well. We argue that it would be beneficial for both students and institutions to work on dropout prevention programs. These programs ought to not only put emphasis on academic factors but also focus on the social integration of local and international students. This could help students get efficient social support when they go through storms in life or struggle with academic difficulties, but could also promote professional socialization of future doctors.

## Data Availability

The data that support the findings of this study are available from the Hungarian Authority of Education but restrictions apply to their availability, which were used under license for the current study, and so are not publicly available. Data are however available from the authors upon reasonable request and with permission of the Hungarian Authority of Education.

## Footnotes

1 1 BA: Bachelor of Arts, BSc: Bachelor of Sciences.

2 2 MA: Masters of Arts, MSc: Masters of Sciences.

3 3 Until the 423/2012. (XII. 29.) government regulation in Hungary came into force in 2020, most fields in Hungarian higher education were accessible without taking advanced level matriculation or by only taking the advanced level exam in one subject of choice. In contrast, to be able to apply to medical fields it was compulsory to take the advanced level exam in the subjects of chemistry and biology.

4 Tuition fees for Hungarian citizens are usually 9000 USD/year.

5 Tuition fees for foreign citizens are 16,000–18,000 USD/year depending on the institution.
